# Field-Scale Spatial Variation of Saline-Sodic Soil and Its Relation with Environmental Factors in Western Songnen Plain of China

**DOI:** 10.3390/ijerph8020374

**Published:** 2011-01-31

**Authors:** Fan Yang, Guangxin Zhang, Xiongrui Yin, Zhijun Liu

**Affiliations:** 1 Key Laboratory of Wetland Ecology and Environment, Northeast Institute of Geography and Agroecology, Chinese Academy of Sciences, 3195 Weishan Road, Changchun 130012, China; E-Mails: zhgx@neigae.ac.cn (G.Z.); yinxiongrui@neigae.ac.cn (X.Y.); 2 Songliao Water Resources Commission, Ministry of Water Resources, Changchun, 130021, China; 3 Dynamic Solutions, LLC, Knoxville, TN 37919, USA; E-Mail: zliu@dsllc.com

**Keywords:** geostatistic, spatial variability, micro-topography, saline-sodic land, western songnen plain

## Abstract

The objectives of this study were to investigate the degree of spatial variability and variance structure of salinization parameters using classical and geostatistical method in Songnen Plain of China, which is one of largest saline-sodic areas in the World, and to analyze the relationship between salinization parameters, including soil salinity content (SC), electrical conductivity (EC), sodium adsorption ratio (SAR), and pH, and seven environmental factors by Pearson and stepwise regression analysis. The environmental factors were ground elevation, surface ponding time, surface ponding depth, and soil moistures at four layers (0–10 cm, 10–30 cm, 30–60 cm, and 60–100 cm). The results indicated that SC, EC, and SAR showed great variations, whereas pH exhibited low variations. Four salinization parameters showed strongly spatial autocorrelation resulting from the compound impact of structural factors. The empirical semivariograms in the four parameters could be simulated by spherical and exponential models. The spatial distributions of SC, EC, SAR and pH showed similar patterns, with the coexistence of high salinity and sodicity in the areas with high ground elevation. By Pearson analysis, the soil salinization parameters showed a significant positive relationship with ground elevation, but a negative correlation with surface ponding time, surface ponding depth, and soil moistures. Both correlation and stepwise regression analysis showed that ground elevation is the most important environmental factor for spatial variation of soil sanilization. The results from this research can provide some useful information for explaining mechanism of salinization process and utilization of saline-sodic soils in the Western Songnen Plain.

## 1. Introduction

Soil salinity and sodicity have become an increasingly acute problem in Songnen Plain in northeast China, one of three largest saline-sodic areas in the World [1]. In addition, the Western Songnen Plain is experiencing the most severe land desertification in North China. The area of saline-sodic soil in Western Songnen Plain is about 2,573,400 ha, approximately 22.62% of its total area [2], and it is increasing at an annual rate of 13,500 ha [2]. The increase in saline-sodic soil leads to serious environmental and social problems, such as the reduction of crop yields and degradation of grasslands [1,3]. Soil salinization has become the main restricting factor for regional sustainable development of grazing and agriculture [2].

Saline and sodic soils often display high spatial variability in soil salinity and sodicity at a field scale [4–8]. It is possibly caused by soil heterogeneity and temporal and spatial variations of external factors. These external factors may include: shallow groundwater, microtopography, seasonal waterlogging, and human activities. The non-homogeneity of soil salinity and sodicity is deemed as one of the most striking characteristic of Western Songnen Plain, and makes it difficult for the development and utilization of saline-sodic soil, ecological reconstruction as well as sustainable development [2], so a quantitative description and explanation of soil salinity heterogeneity is very important for site-specific management in Songnen Plain.

The study on spatial variation of soil salinity and sodicity in Songnen Plain has been focusing on qualitative description and classical statistics analyses [9,10]. However, the classical statistic assumes random variation and cannot account for the spatial correlation. Hence, the classic statistic is insufficient for the interpolation of spatial dependent variables [11].

The objective of this research was to apply geostatistical approaches to determine the degree of spatial variability and variance structure of salinization parameters and evaluate the effects of environmental factors such as micro topography and hydrological elements. The results from this research are of great importance for a better understanding of the mechanism of soil salinization and rational utilization of saline-sodic land resources.

## 2. Materials and Methods

### 2.1. Study Area Description

The experiment is conducted at the Da’an Sodic Land Ecological Experiment Station of the Northeast Institute of Geography and Agroecology, Chinese Academy of Sciences. This region lies in 123º50′27″–123º51′31″ of east longitude and 45º35′58″–45º36′28″ of north latitude. The study area features a temperate continental monsoon climate, with an average annual precipitation of 413.7 mm and an average annual evaporation of 1,696.9 mm. The average annual temperature is 4.7 centigrade with the lowest temperature of −17.7 centigrade in January. Soil is generally frozen from late November and will thaw completely until late May or early June of the next year. The experimental station is surrounded by a low flood plain. Currently, the moderate and mild saline-sodic soils are gradually degraded into moderate and severe saline-sodic soils.

### 2.2. Sampling Procedure and Data Acquisition

**Figure 1.**
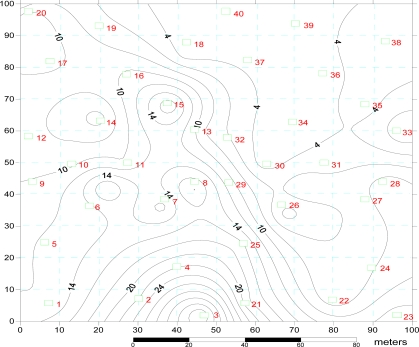
Distribution of measuring points and ground elevation in the experiment site. The numbers in red indicate the randomly selected sampling points. The unit of ground elevation is cm.

A representative saline-sodic area measuring 100 × 100 m was selected within the experimental station for soil sampling. A border (40 cm in height and 40 cm in width) was constructed along the boundary of the experiment area to collect the surface runoff after rainfall events. The experimental area was divided into 10 × 10 m grid squares, and 40 grids were randomly selected as sampling points (Figure 1). The land surface in the area is gently undulating with microtopogrpahy of coexistence of mounds and depressions. The maximum difference in elevation is about 36.3 cm. The grids with the highest elevation (such as sampling points of No.2, No.3, No.4 and No.21) are located in the middle bottom (Figure 1). The grids having the lowest elevation (such as sampling points of No.33, No.34, No.36, No.37, No.39 and No.40) are located in the top right corner (Figure 1). The soil of the experimental area was naturally structured saline-sodic soil. The plant communities in this area mainly include *Phragmites australis*, *Chloris virgata*, *Suaeda glauca* and *Puccinellia tenuiflora.* Typically, *Suaeda glauca* is distributed in the highest elevation areas (mounds), and *Phragmites australis* is distributed in the lowest elevation areas (depressions).

The field experiment was conducted during 1 May to 1 October for three consecutive years (2005 to 2007). The experiment was carried out during May to October because the evapotranporation is the largest in the summer months. Due to the large evapotransporation, the ascending motion of capillary water is generally greater than the descending motion. This facilitates the salt in soil pedon and shallow groundwater to build up on the soil surface [12]. Another reason we selected May to October is that averaged annually about 70% of the rainfall in the study area occurs in July and August. The high-intensity rainfall in the summer months significantly impacted the hydrologic processes including surface runoff, leaching, and surface ponding, which have important effects on the salt buildup and washoff in the soil.

The surface ponding time and surface ponding depth were observed every five days for the no-rainfall period, and every day after the rainfall. At the end of May, July and September, the soil water contents were measured. Soil water contents were observed at four depths of soil layers: 0–10 cm, 10–30 cm, 30–60 cm, and 60–100 cm. Ground elevation was measured by DSN232 balance level.

At the end of September, soil samples were collected to analyze the ions. Soil samples were collected at the depth of 0–10 cm at the 40 sampling points for laboratory analysis. The measured physical and chemical parameters including pH, EC, Na^+^, K^+^, Ca^2+^, Mg^2+^, CO_3_^2−^, HCO_3_^−^,Cl^−^, SO_4_^2−^. All soil samples were air-dried and then passed a 1-mm round-hole sieve for chemical analyses. Soluble salt estimates were based on 1:5 soil-water extracts. The pH and EC of the extracts were determined using a pH meter and a conductivity meter, respectively [13,14]. The concentrations of Na^+^, K^+^, Ca^2+^, and Mg^2+^ were determined by atomic absorption spectrometry (GBC-906AAS). Anion concentrations (CO_3_^2^*^−^*, HCO_3_*^−^*, Cl^−^, SO_4_^2−^) were determined by standard methods [15]. The concentrations of CO_3_^2^*^−^* and HCO_3_*^−^* were determined by a neutral titration method. The concentrations of Cl^−^ were determined by a silver nitrate titration method, and the concentrations of SO_4_^2−^ were determined by a barium sulfate turbidimetric method. Sodium adsorption ratio (SAR) was calculated by the following equation using concentrations of the cations Na^+^, Ca^2+^, and Mg^2+^ [16]:

(1) $$\text{SAR}\;=\;\frac{\left[{\text{Na}}^{+}\right]}{\sqrt{([{\text{Ca}}^{2+}]\;+\;[{\text{Mg}}^{2+}])/2}}$$

### 2.3. Geostatistical Approach

Four soil salinization parameters were analyzed for identifying the outliers and carrying out different transformations such as log normal and square root to ensure a normal distribution. Then semivariogram parameters for each theoretical model such as spherical, exponential, linear, and Gaussian were generated. Selection of the best-fitting model was based on regression statistics such as minimum Residual Sums of Squares (RSS) and maximum determination coefficient (R^2^). The corresponding sill, nugget, and range values of the best-fitting theoretical model were calculated. After selection of the suitable theoretical model and the corresponding semivariogram parameters, spatial variability maps were generated for these four parameters of soil salinization using ordinary kriging. The Pearson correlation and stepwise regression analysis of the data was based on values for salinization parameters and water content of three years.

#### 2.3.1. Semivariogram Modeling

Geostatistics aims at providing quantitative descriptions of natural variables distributed in space and time [17,18]. Based on the regionalized variable theory, geostatistic method assumes that variables in an area exhibit both random and spatially structured properties. The semivariogram is calculated to quantify the spatial structure [19]. The experimental semivariogram is a graphical representation of the mean square variability between two neighboring points of distance h as shown in Equation (2):

(2) $$\gamma (h)\;=\;\frac{1}{2N(h)}\sum_{i=1}^{N(h)}{\left[z(x_{i}\;+\;h)\;-z(x_{i})\right]}^{2}$$

where γ(h) is the semivariogram expressed as a function of the magnitude of the lag distance or separation vector h; N(h) is the number of observation pairs separated by distance h; and z(x_i_) is the regionalized variable at location x_i_.

The experimental semivariogram γ(h) can be fitted by different theoretical models such as spherical, exponential, linear, or Gaussian to determine three semivariogram parameters: the nugget (C_0_), the sill (C_0_ + C), and the range (A_0_) [17].

#### 2.3.2. Ordinary Kriging

Ordinary kriging was used to generate the spatial distribution of these four salinization parameters. The parameter values at the unsampled grids were estimated based on the values at the 40 sampling grids by the ordinary kriging method, which provides the best linear unbiased estimate of a regionalized variable at an unsampled location. Ordinary kriging assumes that the mean of the process is constant and invariant within the spatial domain. A linear combination of available sample values is used for ordinary kriging estimation. Weights, the coefficients of this linear combination, are dependent on two factors: the distance between the sample point and the estimated point and the spatial structure of the variable [20]. This is expressed by Equation (3):

(3) z(χ)=μ+ɛ(χ)

where μ is an unknown constant and generally considered the mean value of the regionalized variable; and z(x) is the value of regionalized variable at any location x with stochastic residual ɛ(x) with zero mean and unit variance.

## 3. Results and Discussion

### 3.1. Distribution Patterns of Salinization Parameters

The descriptive statistics of the spatial distributions of EC, SAR, SC, and pH, are given by Table 1. The calculated statistics included mean, standard deviation (SD), coefficient of variation (CV), minimum, maximum, skewness, kurtosis, and the Kolmogorov–Smirnov test (K–S). From 2005 to 2007, values of EC and SC ranged from 78.2 to 660 μs·cm^−1^, and from 588 to 22,826 mg·kg^−1^, respectively, indicating highly inhomogeneous soil salinity levels. There are also large variations in the observed SAR. The mean values pH ranged from 9.48 to 9.88 during 2005 to 2007, indicating high alkalization level in the study area. CV was the most important factor in describing the variability of a soil property. A CV value lower than 10% indicated low variability while a CV value higher than 100% indicated great variability [1]. The calculated coefficients of variation for EC, SAR, and SC are from 72% to 197%, indicating high spatial variability of EC, SC, and SAR in the experimental area. On contrast, pH showed low spatial variation with CV value of 6–9%. Our experimental results are consistent with other researchers. Gokalp *et al.* reported that EC had the highest CV (125% and 103%), and pH exhibited the lowest CV value (5% and 5%) in 0–30 cm and 30–60 cm soil depths [21]. Cemek *et al.* reported similar CV values for EC (57% in topsoil and 85% in subsoil) and pH (4.7–5%) [22]. For all these three parameters (EC, SC, and SAR) showing high spatial variations, their maximum values are much higher in 2007 than the other two years (Table 1). This might be caused by the relatively lower amount of rainfall in 2007. The observed rainfall during May to October in 2005 and 2006 were 455 and 368.5 mm, respectively, much higher than the 224.5 mm in 2007. The higher evapotransporation deficit in 2007 might cause more salt accumulation in the upper soil by capillary rise.

Frequency distributions and the Kolmogorv–Smirov test for normality showed that EC, SC, and SAR were not normally distributed (*p* < 0.05), with the exception of pH (Table 1). So, mathematical transformations were carried out to convert the data to fit the normal distribution, which is a prerequisite for calibration of the theoretical model and generation of semivariogram parameters and kriged maps. After log-transformation, these soil salinization parameters showed normal distribution. The transformation functions are given by Table 1.

### 3.2. Spatial Structure of Soil Salinization Parameters

**Figure 2.**
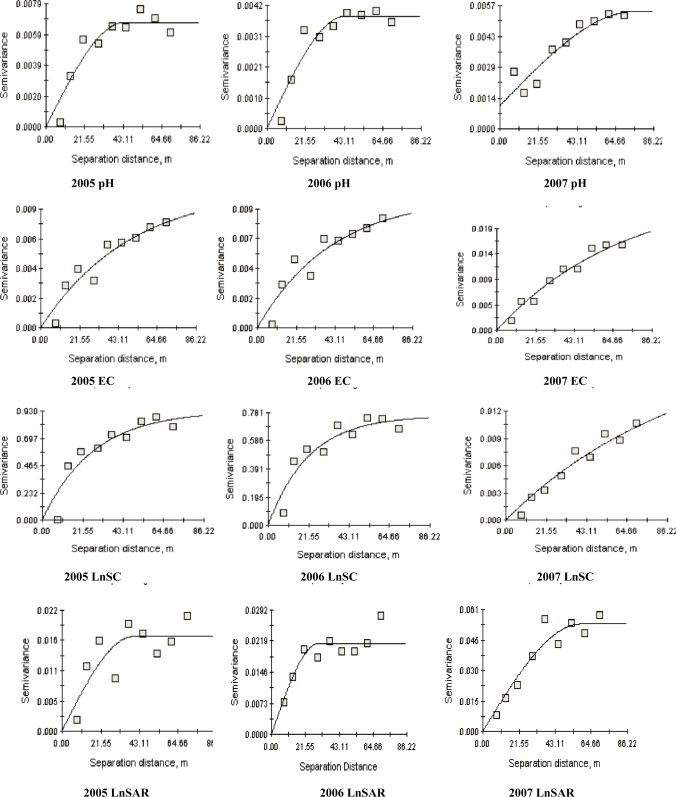
Best-fit semivariograms of soil salinization parameters from 2005 to 2007 year. SC is soil salinity content (mg·kg^−1^); EC is electrical conductivity (μs·cm^−1^); and SAR is sodium adsorption ratio.

A semivariogram for each soil salinization parameter was developed to quantify the spatial variation of soil salinization. The nugget, sill, and range values of the best-fit theoretical models for soil salinization parameters are given in Table 2. The nugget effect is related to the spatial variability in distances shorter than the lowest separation distance between measurements [23]. Meanwhile, the large nugget effect suggested that an additional sampling of these properties at smaller distances and in larger numbers might be needed to detect spatial dependence, and a greater sampling density will result in a more accurate salinity and alkalinity map [22]. The nugget effects of EC, SAR, SC, and pH were low (Table 2). This indicated that four parameters are in big distance, and sampling density and sampling distances were rational. The spatial correlation distances (range) was considered as the distance beyond which observations were not spatially dependent. All values of range were greater than 29.9 m for these four soil salinization parameters. Therefore, all soil salinization parameters had a range value indicating existence of a spatial structure for them (Figure 2). EC range values were from 124.2 to 211.8 m. Our experimental results are consistent with those of other researchers. Range values from 135.8 to 182.5 m were reported by Li *et al.* [24]. It was found by Miyamoto *et al.* that salinity distributions were spatially dependent to a length of 100 m or more [25].

The ratio of nugget to total semivariance, expressed as a percentage, was used to classify spatial dependence: a ratio of <25% indicated strong spatial dependence; between 25% and 75% indicated moderate spatial dependence; and >75% indicated weak spatial dependence [26]. Nugget:sill ratio values for all these four salinization parameters were less than 25%, indicating a strong spatial autocorrelation for four salinization parameters (Table 2). The spherical semivariogram model was found to be the best-fit model for pH and SAR, whereas spatial structures of SC and EC were generally fitted by the exponential model. The results demonstrated that the spatial variations of soil saliniation parameters were mainly affected by structural factors, which might include topography, hydrological and climatic condition [27].

**Figure 3.**
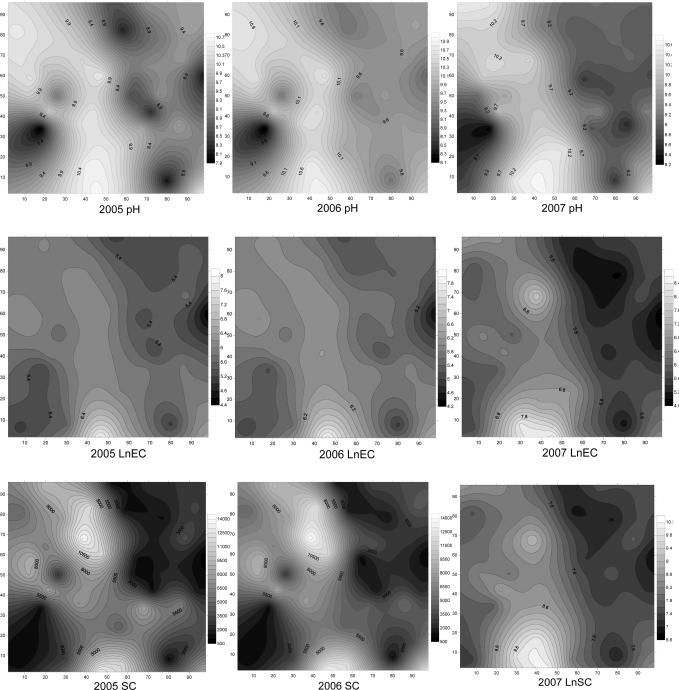

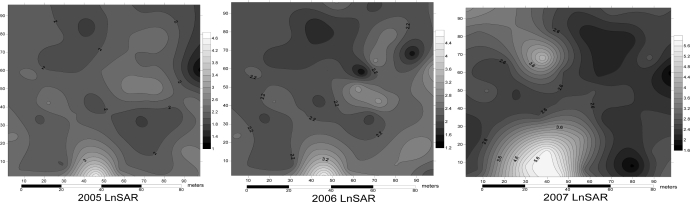
Maps of kriging estimations of four salinization parameters. SC is soil salinity content (mg·kg^−1^); EC is electrical conductivity (μs·cm^−1^); and SAR is sodium adsorption ratio.

Soil salinization parameters exhibited strip and block patterns (Figure 3). Salinization parameters patches were fragmented, indicating a strong spatial variability of soil salinity and sodicity. Based on the Chinese salinization classification, saline soils are classified into five categories: heavy salinized soil (>0.6%), saline soil (0.4–0.6%), moderate salinized soil (0.2–0.4%), light salinized soil (0.1–0.2%), and non-saline soil (<0.1%).

In the study area, high saline and sodic areas co-distribute with non-or low saline and sodic areas, based on Chinese salinzation classification [9]. The spatial distributions of EC, pH, SC, and SAR showed similar patterns (Figure 3). In the experimental area, the high values of soil salinity and sodicity coexisted. The soil salinity and sodicity were intimately dependent on the microtopography and plant distribution: the high salinity and soidicity occurred in the grids located in higher elevation and covered by short grass (*Suaeda glauca* and *Chloris virgata*), while the low salinity and sodicity occurred in the grids located in the lower elevation and covered by high grass (*Phragmites australis*). Similar results have been reported by Douaik *et al.* in that the characterization of low saline areas will be based on samples taken from the lowest locations covered with meadow (waterlogged zone), while the investigation of highly saline areas considers the locations with intermediate elevations and covered with short grass (accumulation zone) when the maximal difference in elevation is 1.76 m [28].

### 3.3. Correlation and Stepwise Regression Analysis

Pearson correlation analysis was conducted to evaluate the effects of environmental factors on these four soil salinization parameters. The investigated environmental factors included ground elevation (H), surface ponding time (d), surface ponding depth (h), and soil moistures at four layers [0–10 cm (W_0–10_), 10–30 cm (W_10–30_), 30–60 cm (W_30–60_), and 60–100 cm (W_60–100_)]. The results of correlation analysis are given by Table 3. The results indicated that the spatial variations of soil salinization were significantly affected by micro-topography and hydrological elements. The ground elevation is significantly positively correlated with these four parameters (*p* < 0.01). Douaik *et al.* also reported that elevation was a major concern in soil salinization [28]. Cemek *et al.* suggested that microtopography in the study area could influence the pattern and magnitude of spatial variability in salinity and alkalinity [22]. These salinization parameters were negatively correlated with h, and d (*p* < 0.01). In the majority of the cases, soil moistures of soil layers were negatively correlated with these four salinization parameters (*p* < 0.05).

The results of stepwise regression analysis also revealed that ground elevation is the most important factor affecting the spatial distribution of salinity and socidity at field scale (Table 4). The regression prediction models with the ground elevation alone can explain the majority of the variations of soil sailinization parameters except pH. For the parameter of pH, the soil moisture at the depth of 10–30 cm is the most important prediction parameters, and ground elevation is the second most important parameter.

**Figure 4.**
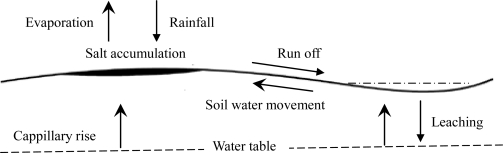
Saline accumulate in subtly undulating landscape.

Minor changes in ground elevation can cause large variations in soil salinity. The higher salinity and soidicity occurred in the grids located in higher elevation, and *vice versa*. This might be explained by the differences in hydrological regime between in the mounds and depressions (Figure 4). In the rainy reason, rainfall runs off the slowly permeable clay into the microrelief depressions in-between the higher elevations. Water then leaches out the salts in the depressions (Figure 4). When there is enough surface runoff to remove salt accumulated in the upper soil in the depression, the soil does not become saline and sodic, but if there is not enough runoff, the soil becomes more saline and sodic than before. If there were surface water ponding, surface water will help lower water table and stop the development of salinity. Because the surface water ponding time is much longer and surface water ponding depth is much deeper in the depressions than in the mounds, which resulted in the higher soil water contents in the depressions than in the mounds. This leads to the lateral soil water movement from the depressions to the adjacent mounds, driven by the gradient of soil water potential between the depressions and mounds, which also transport the salt from the depressions to the mounds (Figure 4). In the dry seasons, the large evaportranspiration will cause the upward movement of water from the shallow groundwater into the upper soil by capillary rise. At the same time, the salt will also be transported to the upper soil by capillary rise. Because of existing of micro-topography and the gradient of soil water potential, much salt was accumulated in the mounds than in the depression.

High levels of sodium restrict waterholding capacity and prevent flocculation of soil particles. The process of soil clay particles gathering together into small aggregates is called flocculation, which allows water to penetrate between the groups of soil particles and provide moisture at deeper depths. During wet conditions the individual clay particles will overlap each other randomly when sodium levels are high enough to prevent flocculation. This will prevent water penetration through the high sodium layer [29]. Consequently the permeability of the upper soil is extremely low with saturated hydraulic conductivity in the mounds because of high sodium level in the mounds. In addition, owing to difference in plant distribution, the infiltration capacity is higher in the depressions than in the mounds. Depressions were covered by *Phragmites australis* due to seasonal waterlogging. The height of *Phragmites australis* ranged from 0.7 to 2.1 m, and the coverage were approximately 70–95%. The roots extend to 60 cm in the soil profile. The advanced root system of *Phragmites australis* can create more active soil pores, which in turn will increase the infiltration capacity. The mounds, however, were covered by saline alkali tolerant plants: *Suaeda glauca* and *Chloris virgata*. These plants are short with heights of 2–8 cm, and are distributed sparsely with coverage of less than 20%. Their root systems can only extend to a soil depth of 3–5 cm. The lower infiltration capacity in the mounds prevented the vertical movement of salt, and caused the accumulation of salt in the upper soil. In the depressions, the salt in the upper soil can relatively easily infiltrate into the deep soil or aquifer. As a result, the mounds were more saline and sodic than the depressions.

## 4. Conclusions

A geostatistical approach was applied to the Western Songnen Plain to investigate the spatial distributions of salinization parameters and the relationship between salinization parameters and environmental factors. The calculated coefficients of variation for EC, SAR, and SC ranged from 72% to 197%, indicating high spatial variation patterns, whereas pH showed a pattern of low spatial variations with a coefficient of variation from 7% to 9%. SC, EC, SAR and pH showed a strong spatial autocorrelation resulting from the compound impact of structural factors. The empirical semivariograms of the four parameters could be simulated by spherical and exponential models.

Correlation and stepwise analysis showed that soil salinization parameters were related in a positive linear fashion to ground elevation, but negatively linearly related to surface ponding time, surface ponding depth, and soil moisture. Ground elevation is the key factor affecting the spatial distribution of soil salinity and sodicity through change hydrological process at the field scale.

## Figures and Tables

**Table 1.** Descriptive statistics on salinization patameters from 2005 to 2007 year.

**SD**	**Parameter**	**Year**	**Min**	**Max**	**Mean**	**CV**	**Ske**	**Kur**	**DP**	**K-S**
0.81	pH	2005	7.87	10.70	9.51	0.09	−0.44	−1.03	N	0.212
0.63		2006	8.06	10.90	9.88	0.06	−0.50	0.23	N	0.411
0.70		2007	8.25	10.60	9.48	0.07	0.10	−1.38	N	0.332

400	EC (μs·cm^−1^)	2005	82.8	2710	392	1.02	5.22	30.50	LN	0.596
411		2006	78	2776	400	1.02	5.18	30.20	LN	0.591
919		2007	123	4730	660	1.39	3.51	12.80	LN	0.764

3,833	SC (mg·kg^−1^)	2005	588	13,616	5,212	0.74	5.22	30.50	N	0.596
3,556		2006	1,134	14,687	5,366	0.72	5.18	30.20	N	0.591
5,105		2007	874	22,826	4,068	1.25	2.93	8.07	LN	0.759

12.60	SAR	2005	3.09	86.90	10.60	1.19	5.98	37.00	LN	0.248
13.40		2006	3.91	91.10	12.00	1.12	5.51	32.90	LN	0.385
70.90		2007	5.55	306.00	35.90	1.97	3.26	9.66	LN	0.060

Note: EC is electrical conductivity; SC is soil salinity content; SAR is sodium adsorption ratio; Min is the minimum value; Max is the maximum value; Mean is the average value; SD is the standard deviation; CV is the calculated coefficient of variation; Ske is the calculated skewness; Kur is the calculated Kurtosis; K-S is the coefficient of Kolmogorav-Smirnow; DP is the distribution pattern; N is normal distribution; and LN is normal distribution after logarithmic transformation.

**Table 2.** Summary of best-fit models for salinization parameters.

**Parameter**	**Year**	**Best-fit model**	**Nugget, C_0_**	**Sill, C_0_+ C**	**Range (m) A_0_**	**C_0_/(C_0_+ C) (%)**	**R^2^**	**RSS**
pH	2005	Spherical	0.00001	0.007	43.6	0.200	0.875	0.0000007
pH	2006	Spherical	0.00001	0.004	44.0	0.300	0.891	0.0000015
pH	2007	Spherical	0.00107	0.005	76.2	20.000	0.888	0.0000018
LnEC	2005	Exponential	0.00001	0.010	150.0	0.100	0.906	0.0000044
LnEC	2006	Exponential	0.00032	0.011	124.0	0.100	0.884	0.0000061
LnEC	2007	Exponential	0.00000	0.026	212.0	0.000	0.965	0.0000083
SC	2005	Exponential	0.00100	0.930	81.0	0.100	0.862	0.0870000
SC	2006	Exponential	0.00100	0.761	64.8	0.100	0.860	0.0510000
LnSC	2007	Exponential	0.00001	0.019	285.0	0.100	0.960	0.0000048
LnSAR	2005	Spherical	0.00013	0.017	39.6	0.800	0.606	0.0001059
LnSAR	2006	Spherical	0.00000	0.021	29.9	0.000	0.756	0.0006431
LnSAR	2007	Spherical	0.00010	0.053	58.4	0.002	0.914	0.0002494

Note: SC is soil salinity content (mg·kg^−1^); EC is electrical conductivity (μs·cm^−1^); SAR is sodium adsorption ratio; R^2^ is the determination coefficient; and RSS is residual sums of squares.

**Table 3.** Pearson analysis between salinization parameters and environmental factors.

**Parameter**	**H**	**h**	**d**	**W_0–10_**	**W_10–30_**	**W_30–60_**	**W_60–100_**
EC	0.811**	−0.46**	−0.486**	−0.273	−0.387*	−0.510**	−0.379*
pH	0.592**	−0.559**	−0.572**	−0.532**	−0.633**	−0.468**	−0.529**
SAR	0.744**	−0.324*	−0.353*	−0.165	−0.249	−0.400*	−0.250
SC	0.688**	−0.638**	−0.624**	−0.589**	−0.557**	−0.559**	−0.351*

Note: SC is soil salinity content (mg·kg^−1^); EC is electrical conductivity (μs·cm^−1^); SAR is sodium adsorption ratio; H is ground elevation(cm); h is surface ponding depth(cm); d is surface ponding time(day); W_0–10_, W_10–30_, W_30–60_ and W_60–100_ are the soil moistures at 0–10, 10–30, 30–60, and 60–100 cm, respectively;

** p < 0.01;

* p < 0.05.

**Table 4.** Regression model between salinization parameters and environmental factors.

**Regression model**	**R^2^**	**Sig**
EC = 60.809H − 108.559	0.658	0.000
EC = 82.821H + 13.710d − 613.38	0.713	0.000
SC = 375.272H + 2000.542	0.446	0.000
SC = 279.3H − 32716.3W_60–100_ + 10779.1	0.531	0.000
SAR = 1.266H − 3.199	0.542	0.000
SAR = 2.037H + 0.481d − 20.896	0.687	0.000
pH = −7.227W_10–30_ + 11.361	0.401	0.000
pH = 0.034H − 5.108W_10–30_ + 10.51	0.498	0.000

Note: SC is soil salinity content (mg·kg^−1^); EC is electrical conductivity (μs·cm^−1^); SAR is sodium adsorption ratio; R^2^ is the determination coefficient, and Sig is the statistic significance level.
